# Meropenem/Vaborbactam Plus Aztreonam as a Possible Treatment Strategy for Bloodstream Infections Caused by Ceftazidime/Avibactam-Resistant *Klebsiella pneumoniae*: A Retrospective Case Series and Literature Review

**DOI:** 10.3390/antibiotics11030373

**Published:** 2022-03-10

**Authors:** Alessandra Belati, Davide Fiore Bavaro, Lucia Diella, Nicolò De Gennaro, Francesco Di Gennaro, Annalisa Saracino

**Affiliations:** Clinic of Infectious Diseases, University Hospital Policlinico, University of Bari, Piazza Giulio Cesare n. 11, 70124 Bari, Italy; alessandra.belati@hotmail.it (A.B.); diella.lucia@gmail.com (L.D.); nico84degennaro@gmail.com (N.D.G.); francesco.digennaro1@uniba.it (F.D.G.); annalisa.saracino@uniba.it (A.S.)

**Keywords:** carbapenem-resistant Gram-negative, carbapenem-resistant *Klebsiella pneumoniae*, metallo-beta-lactamase carbapenemases, avibactam resistance, meropenem/vaborbactam, aztreonam

## Abstract

Objectives: The aim of this study was to describe our experience of a combination treatment including meropenem/vaborbactam (M/V) plus aztreonam (ATM) for bloodstream infections (BSIs) due to ceftazidime/avibactam-resistant *Klebsiella pneumoniae* (CAZ/AVI-R-*Kp*), for which gene typing was not available at the time the blood culture (BC) results were obtained. Methods: Between 20 July and 22 August 2021, in our hospital laboratory, the molecular test for carbapenemase gene typing was not available. All Gram-negative bloodstream infections were recorded, and characteristics of patients were analysed. Among them, three patients had positive BCs for CAZ/AVI-R-*Kp*, and the empirical therapy was switched to M/V plus ATM pending phenotypic testing of sensitivity to M/V. Therapy was subsequently targeted on the basis of the results of this test. Results: KPC and NDM represent the most prevalent carbapenemases in our polyclinic. Three patients with CAZ/AVI-R-*Kp* sepsis were treated with M/V plus ATM not knowing the carbapenemase gene. Two had an NDM-*Kp* infection for which, upon obtaining the result of sensitivity to M/V, combination therapy was maintained. The third had KPC-*Kp* infection for which ATM was discontinued, after the acquisition of an antibiogram reporting full sensitivity to M/V (MIC = 0.25 mg/L). One patient with NDM-*Kp* infection died due to complications of the underlying disease for which he was hospitalised. Conclusions: Meropenem/vaborbactam plus ATM and subsequent de-escalation could represent a possible therapeutic strategy in severe CAZ/AVI-R-*Kp* infections when carbapenemase gene typing is not rapidly available.

## 1. Introduction

Carbapenem resistance represents a major concern worldwide. The World Health Organization identified a list of priority pathogens for which new antibiotics are urgently needed, including carbapenem-resistant *Acinetobacter baumannii* (CRAB), carbapenem-resistant *Pseudomonas aeruginosa* (CR-Pa) and carbapenem-resistant *Enterobacterales* (CRE). Among CRE, *Klebsiella pneumoniae* (*K. pneumoniae*) is the most commonly found and represents a frequent cause of hospital-acquired infections, burdened by great morbidity and mortality [1].

Carbapenem resistance in Gram-negative bacteria (GNB) results from two main mechanisms: (i) acquisition and expression of carbapenemase genes, encoding for enzymes that hydrolyse carbapenems, and (ii) expression of porin/efflux pumps in combination with overexpression of β-lactamases with weak affinity for carbapenems [2].

Genes for carbapenemases are classified according to Ambler’s classification into four categories, and those most widespread in the world are KPC-type enzymes, metallo-β-lactamases (MBLs) (NDM, VIM, IMP) and OXA-48-type enzymes, with a different pattern of sensitivity to antibiotics [2].

MBL-producing *K. pneumoniae* (MBL-*Kp*) are resistant to ceftazidime/avibactam, and colistin still represents the backbone of the therapy. In this setting, colistin has been extensively used as a therapeutic option against CRE; however, the wide use of this molecule as a last resort has caused the emergence of multiple mechanisms of resistance to colistin among carbapenem-resistant *Enterobacteriaceae*, including the spread of the mcr-1 plasmid [3,4]. Indeed, multiple mechanisms of resistance to colistin have been identified, including also changes in the outer bacterial membrane [5]. Therefore, according to the current literature, high-dose and combination strategies including the new β-lactam/β-lactamase inhibitors should be considered for treatment of infections caused by CRE [6]. For instance, aztreonam (ATM) plus avibactam showed a valid alternative strategy to treat infections due to MBL-*Kp*, thanks to the preserved activity of ATM on MBLs and the activity of avibactam on concomitant co-expressed β-lactamases (ESBLs, KPC and other cephalosporinases) [7].

On the contrary, ceftazidime/avibactam (CAZ/AVI), alone or in combination, represents the first choice for KPC-producing *K. pneumoniae* (KPC-*Kp*), although emerging resistance to avibactam poses a therapeutic challenge [8].

Recently, new antimicrobials such as cefiderocol, meropenem/vaborbactam (M/V) and imipenem/cilastatin/relebactam have been approved for KPC-*Kp* with limited indications [9], and real-life data are still lacking [10,11,12,13].

In this scenario, a rapid genotypic antibiogram becomes crucial for gene detection and appropriate antibiotic selection, but if it is not available, phenotypic testing is time-consuming and clinicians cannot wait for results before starting an appropriate therapy [14].

Meropenem/vaborbactam plus ATM has been proposed as an effective treatment option for both MBL-*Kp* and KPC-*Kp* while waiting for phenotypic tests, but studies are limited to in vitro studies [15].

Herein, we describe three cases for which the combination M/V plus ATM has been used for CAZ/AVI-R-*Kp,* due to the unavailability of molecular tests in our hospital laboratory, pending the phenotypic results for new antimicrobials.

## 2. Methods

### 2.1. Design of the Study

This was an observational retrospective study, conducted in a tertiary care hospital in Bari, Italy. Between 20 July and 25 August 2021, in our hospital laboratory, the molecular test for carbapenemase gene typing was not available. In this period, all GNB bloodstream infections (BSIs) were recorded. Patient and microbiological data were analysed. All patients were followed up for 30 days after their BSI episode.

### 2.2. Microbiologic Testing

Blood cultures (BCs) were processed by BactAlert System (Biomerieux Inc., Marcy l’Etoile, France), isolate identification and antibiograms were performed using VITEK-MS (Biomerieux Inc., Marcy l’Etoile, France) and for meropenem/vaborbactam, an E-test was performed (ETEST^®^ Meropenem/Vaborbactam, BioMérieux Inc., Marcy l’Etoile, France). Minimum inhibitory concentrations (MICs) were classified according to breakpoints established by European Committee on Antimicrobial Susceptibility Testing guidelines (EUCAST) [16].

### 2.3. Antimicrobial Treatment Strategy

Targeted therapy was decided by an infectious diseases specialist on the basis of the phenotypic profile of the blood isolate, blinded to the study. The antimicrobial therapy was interrupted after 7–10 days according to our internal protocol, which provides for the execution of follow-up BCs after 48 h from the start of the targeted antibiotic therapy, clinical (absence of fever for at least 48 h and absence of other sepsis signs) and laboratory (procalcitonin < 2 or reduced by 80% compared to baseline) improvement and the absence of deep foci of infection. Antibiotics were prescribed according to their PK/PD: all beta-lactams were prescribed in an extended infusion; aminoglycosides were prescribed in single-dose administration for a short period.

### 2.4. Outcomes

The main outcomes were 30-day all-cause mortality, and microbiological eradication on the 14th day.

### 2.5. Statistical Analysis

Standard descriptive statistics were used to summarise data, such as the mean, median, interquartile range and percentage. The MedCalc statistical software package, version 18.2.1 (MedCalc Software, Ostend, Belgium), was used for all statistical analyses.

### 2.6. Ethical Approval

Moreover, this study was performed with the formal approval of our ethical committee (study number: 6527) and in accordance with the Declaration of Helsinki and national and institutional standards. The patients provided written informed consent (available from the corresponding author) for the use of their data for research purposes. Finally, data were previously pseudo-anonymised, according to the requirements set by the Italian Data Protection Code (leg. Decree 196/2003) and the European General Data Protection Regulation (GDPR 2016/679).

## 3. Results

Overall, 23 BSIs were recorded. Twelve were caused by Gram-positive pathogens and were excluded. Eleven were caused by GNB.

Three patients were severely immunocompromised due to cardiac transplantation, kidney transplantation and drug-induced neutropenia, retrospectively. Two patients had a brain tumor.

**Figure 1:**
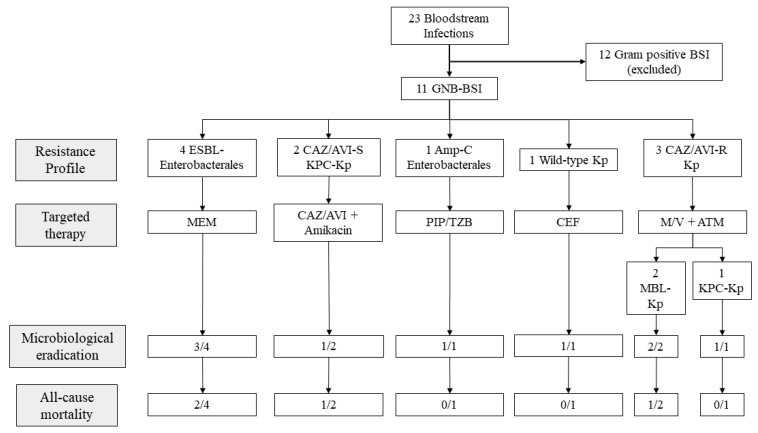
Etiologic characteristics, targeted treatment, microbiological eradication and all-cause mortality of all GNB-BSIs. Legend: ATM = aztreonam; BSI = bloodstream infection; CAZ/AVI = ceftazidime/avibactam; CEF = cefepime; ESBL = extended-spectrum β-lactamases; Kp = *Klebsiella pneumoniae*; MEM = meropenem; M/V meropenem/vaborbactam; PIP/TZB = piperacillin/tazobactam; R = resistant; S = sensitive.

The etiologic characteristics, targeted treatment, microbiological eradication and all-cause mortality of all GNB-BSIs are summarised in Figure 1.

All patients received an empirical therapy on the suspicion of sepsis, according to patient characteristics, comorbidities, risk factors for multidrug-resistant organisms and known colonisation, but in 5/11 patients, it was inappropriate. One patient received a de-escalation targeted therapy with cefepime. For one patient, piperacillin/tazobactam was confirmed as a targeted therapy.

Three patients received ATM plus M/V, since the *K. pneumoniae* strains isolated were all resistant to CAZ/AVI in the phenotypic antibiogram (Table 1). The cases are described below.

All but two patients achieved microbiological eradication, investigated by follow-up blood cultures. Nevertheless, four patients died: two for failure to eradicate infection, one for a breakthrough infection caused by *Acinetobacter baumannii* and one for gastrointestinal massive bleeding.

### 3.1. Case 1

The first patient was a 55-year-old male. He was admitted to the Cardiac Surgery Department for acute cardiac failure due to a dilatative cardiomyopathy. Urgent cardiac transplantation was performed. After 10 days, he presented with fever and hypotension. Blood cultures were performed and empiric therapy with meropenem plus daptomycin was started. After 48 h, according to the antibiogram (Table 1, strain 1), daptomycin was discontinued, and meropenem was switched to M/V 2/2 gr in a 30 min infusion as the loading dose, followed by 2/2 gr in a 3 h infusion tid plus ATM 2 gr in a 30 min infusion as the loading dose, and then 2 gr in a 3 h infusion tid, and the E-test for M/V was performed. Twenty-four hours later, the M/V E-test resulted in being sensitive for M/V. Consequently, concluding from the phenotypic antibiogram that the encoded gene was KPC, ATM was discontinued. The patient recovered and the antibiotic therapy was stopped on the 7th day, according to our internal protocol. The patient was transferred to the rehabilitation center of our hospital, and he is still in follow-up. No recrudescence of *K. pneumoniae* infection was recorded up to 30 days from the described infection.

### 3.2. Case 2

The second patient was a 53-year-old female, with underlying hypertension and von Willebrand disease. She was admitted to the General Surgery Department for diverticulitis complicated by intestinal perforation, and she underwent sigmoidectomy. On the 9th post-operative day, she had fever and wound dehiscence. A computed tomography scan of her abdomen was performed, with evidence of multiple intrabdominal collections. Blood cultures were performed, and an empirical therapy with meropenem plus teicoplanin and tigecycline was started by the surgery team. After 48 h, BCs turned positive for ceftazidime/avibactam-resistant *K. pneumoniae* (Table 1, strain 2). The infectious diseases consultant started the antimicrobial therapy with M/V 2/2 gr in a 30 min infusion as the loading dose, followed by 2/2 gr in a 3 h infusion tid plus ATM 2 gr in a 30 min infusion as the loading dose, and then 2 gr in a 3 h infusion tid and fosfomycin 6 gr in a 90 min infusion tid. Twenty-four hours later, the M/V E-test resulted in being resistant for M/V; therefore, on suspicion of the MBL enzyme, the combination therapy was maintained, according to clinical improvement. Intra-abdominal collections were drained, and antibiotic therapy was discontinued after three weeks. Unfortunately, the patient died 7 days after because of gastrointestinal massive bleeding.

### 3.3. Case 3

The third patient was a 52-year-old female with a history of neurosurgical peritoneal ventricle shunt surgery for intracranial hemorrhage. She was admitted to the Emergency Department for fever and confusion. Blood cultures were performed, and empiric therapy with piperacillin/tazobactam plus fosfomycin was started. After 48 h, BCs turned positive for ceftazidime/avibactam-resistant *K. pneumoniae* (Table 1, strain 3). The patient was still febrile, with elevated inflammation biomarkers. The infectious diseases consultant started the antimicrobial therapy with M/V 2/2 gr in a 30 min infusion as the loading dose, followed by 2/2 gr in a 3 h infusion tid plus ATM 2 gr in a 30 min infusion as the loading dose, and then 2 gr in a 3 h infusion tid. Twenty-four hours later, the M/V E-test resulted in being resistant for M/V; therefore, the combination therapy was maintained, on suspicion of MBL-encoding *K.pneumoniae* infection. The patient was followed up and hospitalised in the Internal Medicine Department. The antibiotic therapy was discontinued after 9 days according to our internal protocol. The patient was discharged and followed up. No recrudescence of infection was recorded up to 90 days from the described infection.

## 4. Discussion

The aim of this study was to provide a preliminary proof of concept regarding the empirical use of M/V plus ATM for the treatment of infections caused by ceftazidime/avibactam-resistant *Enterobacterales*, pending the result of carbapenemase identification, in the context of the high prevalence of KPC and MBL resistance genes. Indeed, this association could provide an effective therapy in the case of confirmed infection by MBL-producing *Enterobacterales*, or may be subsequently de-escalated to M/V alone in the case of confirmed KPC-producing *Enterobacterales*.

To date, carbapenem-resistant *Enterobacterales* are still a serious clinical challenge, with limited treatment options burdened by the emergence of several carbapenemases. In fact, for KPC-producing *Enterobacterales*, new antimicrobials have recently been commercialised, including M/V and imipenem/relebactam/cilastatin, which showed important activity in deep site infections and could overcome ceftazidime/avibactam resistance [17]. In general, the phenotypic sensitivity in vitro to CAZ/AVI may be predictive of a KPC-producing *Enterobacterales*; nevertheless, the emerging resistance to ceftazidime/avibactam, for instance, caused by the mutation of KPC-3 poses a challenge in predicting the resistance gene if the genotypic test is not available [8]. In this case, it is necessary to perform additional phenotypic tests that are time-consuming, with the risk of delaying appropriate therapy, particularly in critically ill patients.

In addition, avibactam resistance may also be caused by the presence of MBL resistance genes, posing a serious clinical challenge in the prescription of an initial empirical therapy. In fact, the treatment of MBL-producing *Enterobacterales* is currently based on colistin combination therapies [18], although the efficacy and safety of these regimens are unsatisfactory. Therefore, several alternative treatments have been explored, including the association of ATM plus CAZ/AVI, but real-life data are still limited.

Mauri et al. recently published a review focusing on the in vitro and in vivo efficacy of ATM plus CAZ/AVI, including 2209 Gram-negative strains tested in vitro for the ATM plus CAZ/AVI combination, finding a high antimicrobial activity of ATM (MIC ≤ 4 mg/L) when combined with avibactam in 80% of MBL-producing *Enterobacterales*, 85% of *Stenotrophomonas* spp. and 6% of MBL-producing *Pseudomonas* spp. Clinical data were available for 94 patients, of whom 64 (83%) had a BSI. Death occurred in 19% of cases [19].

In a prospective observational study, Falcone et al. analysed 102 patients infected with MBL-producing enterobacteria, of whom 52 were treated with ATM plus CAZ/AVI and 50 with other active antibiotics. The ATM plus CAZ/AVI combination was associated with lower 30-day mortality, lower clinical failure at day 14 and a shorter length of stay [7,15].

However, in the context of the high diffusion of *Enterobacterales* both encoding KPC but resistant to CAZ/AVI and producing MBL, the empirical association of ATM plus CAZ/AVI may be suboptimal, pending the definitive genotyping results. Accordingly, on the basis of the current literature, we decided to include the empirical association of ATM plus M/V for the initial therapy of ceftazidime/avibactam-resistant *Enterobacterales* in our internal protocol, due to the unavailability of rapid genotyping/phenotyping tests and the concurrent high prevalence of ceftazidime/avibactam-resistant KPC and MBL.

Indeed, different studies have already investigated the in vitro activity of ATM plus vaborbactam and other β-lactamase inhibitors (summarised in Table 2). According to Avery et al., the use of synergy tests with antibiotic gradient diffusion strips supplies a phenotypic profile distinguished by sizeable zones of inhibited bacterial growth defined as the “zone of hope” that may predict the genotypic profile (serine β-lactamases or MBLs) in the absence of rapid molecular diagnostic systems [20].

However, to the best of our knowledge, this is the first work in which the M/V plus ATM combination has been used to treat patients with MBL-*Kp* infections in a “real-life” scenario.

Notably, OXA-like carbapenemases represent a concern of this treatment strategy, since M/V is not effective against this type of enzyme [24]; however, in our centre, OXA-like enzymes are rarely reported, so this combination represents, in our experience, a viable option pending the definitive genotypic and phenotypic results. In any case, an important limitation of this work should be noted: the unavailability of rapid gene detection on blood cultures that did not allow a faster targeted therapy within the first hours from pathogen identification.

In conclusion, in our small experience, the combination of ATM plus M/V was effective in the microbiological and clinical cure of patients with MBL infection, while allowing effective therapy for CAZ/AVI-RKPC-*Kp* infection.

## 5. Conclusions

The combination of ATM plus M/V could be a valid option in settings where OXA-like enzyme circulation is rare, representing a valid treatment option for both KPC-producing and MBL-producing *Enterobacterales* pending the genotyping/phenotyping results.

## Figures and Tables

**Table 1:** Antimicrobial susceptibility test of three *Klebsiella pneumoniae* strains resistant to avibactam.

	Strain 1 (KPC-*Kp*)	Strain 2 (MBL-*Kp*)	Strain 3 (MBL-*Kp*)
Antimicrobial	MIC	MIC	MIC
Amikacin	≤1 **	4 **	NA
Amoxicillin/Clavulanate	>16	>16	>16
Cefepime	>16	>16	>16
Cefotaxime	>32	>32	>32
Ceftazidime	>32	>32	>32
Ceftazidime/Avibactam	>8	>8	>8
Ceftolozane/Tazobactam	>8	>8	>8
Ciprofloxacin	>2	>2	>2
Colistin *	2	>2	>2
Gentamycin	2	8	8
Imipenem	>8	>8	>8
Meropenem	>8	>8	>8
Piperacilline/Tazobactam	>64	>64	>64
Tobramycin	8	8	8
Trimetropim/Sulphametoxazole	>160	>160	>160
Meropenem/Vaborbactam *	0.25 **	128	128

Legend: * All antibiotics but M/V and colistin were tested with Vitek-MS. Meropenem/vaborbactam was tested with an E-test. Colistin was tested with the broth microdilution method. M/V = meropenem/vaborbactam; NA = not available; ** = interpretation: sensitive.

**Table 2:** Studies about the synergistic effect of aztreonam plus different β-lactamase inhibitors.

Reference	Study	Year	N. of Strains	Microbiological Test	Combination Tested	Results
[15]	Biagi et al.	2019	8	Broth microdilution	ATM + CAZ/AVI	** 87,5%ATM MIC reduction
					ATM + M/V	** 75% ATM MIC reduction
[20]	Avery et al.	2019	15	E-test	ATM + CAZ/AVI	Median ZOH 75.4
					ATM + M/V	Median ZOH 23.5
[21]	Biagi et al.	2020	47	Broth microdilution	ATM + AVI	** 98% MIC restored
					ATM + CLA	** 61% MIC restored
					ATM + REL	** 71% MIC restored
					ATM + VAB	** 15% MIC restored
[22]	Maraki et al.	2021	40	E-test	ATM + CAZ/AVI	** 97.5% MIC reduction
					ATM + M/V	** 97.5% MIC reduction
					ATM + I/R	** 72.5% MIC reduction
[23]	Morroni et al.	2021	9	Broth microdilution	ATM + CLA	0% MIC reduction ≤ 4 *
					ATM + SUL	0% MIC reduction ≤ 4 *
					ATM + TZB	** 11% MIC reduction ≤ 4 *
					ATM + VAB	** 22% MIC reduction ≤ 4 *
					ATM + AVI	** 44% MIC reduction ≤ 4 *
					ATM + REL	** 44% MIC reduction ≤ 4 *
					ATM + ZID	** 100% MIC reduction ≤ 4 *

Legend: ATM = aztreonam; AVI = avibactam; CAZ/AVI = ceftazidime/avibactam; CLA = clavulanate; I/R = imipenem/relebactam/cilastatin; MIC = minimal inhibitory concentration; REL = relebactam; VAB = vaborbactam; SUL = sulbactam; TZB = tazobactam; ZID = zidebactam; ZOH = zone of hope. * We have extrapolated these results from the cited work. ** Percentages refer to the number of strains which showed MIC restoration.

## Data Availability

The dataset is available from the corresponding author on reasonable request.

## References

[1] World Health Organization (2017). Prioritization of Pathogens to Guide Discovery, Research and Development of New Antibiotics for Drug Resistant Bacterial Infections, including Tuberculosis. http://www.who.int/medicines/areas/rational_use/prioritization-of-pathogens/en/.

[2] Nordmann P., Dortet L., Poirel L. (2012). Carbapenem resistance in Enterobacteriaceae: Here is the storm!. Trends Mol. Med..

[3] Gogry F.A., Siddiqui M.T., Haq Q.M.R. (2019). Emergence of mcr-1 conferred colistin resistance among bacterial isolates from urban sewage water in India. Environ. Sci. Pollut. Res..

[4] Gogry F.A., Siddiqui M.T., Sultan I., Haq Q.M.R. (2021). Current Update on Intrinsic and Acquired Colistin Resistance Mechanisms in Bacteria. Front. Med..

[5] Gogry F.A., Siddiqui M.T., Sultan I., Husain F.M., Al-Kheraif A.A., Ali A., Haq Q.M.R. (2022). Colistin Interaction and Surface Changes Associated with mcr-1 Conferred Plasmid Mediated Resistance in *E. coli* and *A. veronii* Strains. Pharmaceutics.

[6] Sheu C.C., Chang Y.T., Lin S.Y., Chen Y.H., Hsueh P.R. (2019). Infections Caused by Car-bapenem-Resistant Enterobacteriaceae: An Update on Therapeutic Options. Front. Microbiol..

[7] Falcone M., Daikos G.L., Tiseo G., Bassoulis D., Giordano C., Galfo V., Leonildi A., Tagliaferri E., Barnini S., Sani S. (2021). Efficacy of Ceftazidime-avibactam Plus Aztreonam in Patients with Bloodstream Infections Caused by Metallo-β-lactamase-Producing Enterobacterales. Clin. Infect. Dis..

[8] Haidar G., Clancy C.J., Shields R.K., Hao B., Cheng S., Nguyen M.H. (2017). Mutations in blaKPC-3 That Confer Ceftazidime-Avibactam Resistance Encode Novel KPC-3 Variants That Function as Extended-Spectrum β-Lactamases. Antimicrob. Agents Chemother..

[9] Yahav D., Giske C.G., Grāmatniece A., Abodakpi H., Tam V.H., Leibovici L. (2020). New β-Lactam-β-Lactamase Inhibitor Combinations. Clin. Microbiol. Rev..

[10] Bavaro D.F., Belati A., Diella L., Stufano M., Romanelli F., Scalone L., Stolfa S., Ronga L., Maurmo L., Dell’Aera M. (2021). Cefiderocol-Based Combination Therapy for “Difficult-to-Treat” Gram-Negative Severe Infections: Real-Life Case Series and Future Perspectives. Antibiotics.

[11] Bavaro D.F., Romanelli F., Stolfa S., Belati A., Diella L., Ronga L., Fico C., Monno L., Mosca A., Saracino A. (2021). Recurrent neurosurgical site infection by extensively drug-resistant P. aeruginosa treated with cefiderocol: A case report and literature review. Infect. Dis..

[12] Alosaimy S., Lagnf A.M., Morrisette T., Scipione M.R., Zhao J.J., Jorgensen S., Mynatt R., Carlson T.J., Jo J., Garey K.W. (2021). Real-world, Multicenter Experience with Meropenem-Vaborbactam for Gram-Negative Bacterial Infections Including Carbapenem-Resistant Enterobacterales and Pseudomonas aeruginosa. Open Forum Infect. Dis..

[13] Shields R.K., McCreary E.K., Marini R.V., Kline E.G., Jones C.E., Hao B., Chen L., Kreiswirth B.N., Doi Y., Clancy C.J. (2020). Early Experience with Meropenem-Vaborbactam for Treatment of Carbapenem-resistant Enterobacteriaceae Infections. Clin. Infect. Dis. Off. Publ. Infect. Dis. Soc. Am..

[14] Poirel L., Walsh T.R., Cuvillier V., Nordmann P. (2011). Multiplex PCR for detection of acquired carbapenemase genes. Diagn. Microbiol. Infect. Dis..

[15] Biagi M., Wu T., Lee M., Patel S., Butler D., Wenzler E. (2019). Searching for the Optimal Treatment for Metallo- and Serine-β-Lactamase Producing Enterobacteriaceae: Aztreonam in Combination with Ceftazidime-avibactam or Meropenem-vaborbactam. Antimicrob. Agents Chemother..

[16] The European Committee on Antimicrobial Susceptibility Testing (2021). Breakpoint Tables for Interpretation of MICs and Zone Diameters. Version 11.0. http://www.eucast.org.

[17] Bassetti M., Poulakou G., Ruppe E., Bouza E., Van Hal S.J., Brink A. (2017). Antimicrobial resistance in the next 30 years, humankind, bugs and drugs: A visionary approach. Intensive Care Med..

[18] Soman R., Bakthavatchalam Y.D., Nadarajan A., Dwarakanathan H.T., Venkatasubramanian R., Veeraraghavan B. (2021). Is it time to move away from polymyxins? Evidence and alternatives. Eur. J. Clin. Microbiol. Infect. Dis..

[19] Mauri C., Maraolo A.E., Di Bella S., Luzzaro F., Principe L. (2021). The Revival of Aztreonam in Combination with Avibactam against Metallo-β-Lactamase-Producing Gram-Negatives: A Systematic Review of in vitro Studies and Clinical Cases. Antibiotics.

[20] Avery L.M., Mullane E.M., Nicolau D.P. (2020). Evaluation of the in vitro activity of WCK 5222 (cefepime/zidebactam) and currently available combination therapies against single- and double-carbapenemase producing *Enterobacteriaceae*: Expanding the zone of hope. Int. J. Antimicrob. Agents.

[21] Biagi M., Lamm D., Meyer K., Vialichka A., Jurkovic M., Patel S., Mendes R.E., Bulman Z.P., Wenzler E. (2020). Activity of Aztreonam in Combination with Avibactam, Clavulanate, Relebactam, and Vaborbactam against Multidrug-Resistant Stenotrophomonas maltophilia. Antimicrob. Agents Chemother..

[22] Maraki S., Mavromanolaki V.E., Moraitis P., Stafylaki D., Kasimati A., Magkafouraki E., Scoulica E. (2021). Ceftazidime-avibactam, meropenen-vaborbactam, and imipenem-relebactam in combination with aztreonam against multidrug-resistant, metallo-β-lactamase-producing *Klebsiella pneumoniae*. Eur. J. Clin. Microbiol. Infect. Dis..

[23] Morroni G., Bressan R., Fioriti S., D’Achille G., Mingoia M., Cirioni O., Di Bella S., Piazza A., Comandatore F., Mauri C. (2021). Antimicrobial Activity of Aztreonam in Combination with Old and New β-Lactamase Inhibitors against MBL and ESBL Co-Producing Gram-Negative Clinical Isolates: Possible Options for the Treatment of Complicated Infections. Antibiotics.

[24] Doi Y. (2019). Treatment Options for Carbapenem-resistant Gram-negative Bacterial Infections. Clin. Infect. Dis..

